# MicroRNA signature of cis-platin resistant vs. cis-platin sensitive ovarian cancer cell lines

**DOI:** 10.1186/1757-2215-4-17

**Published:** 2011-09-22

**Authors:** Smriti Kumar, Arooshi Kumar, Parag P Shah, Shesh N Rai, Siva K Panguluri, Sham S Kakar

**Affiliations:** 1 James Graham Brown Cancer Center, University of Louisville, Louisville, KY 40202, USA; 2 Massachusetts Institute of Technology (MIT), Boston, MA, USA; 3 Department of Physiology and Biophysics, University of Louisville, Louisville, KY 40202, USA; 4 Anatomical Sciences and Neurobiology, University of Louisville, Louisville, KY 40202, USA

## Abstract

**Background:**

Ovarian cancer is the leading cause of death from gynecologic cancer in women worldwide. According to the National Cancer Institute, ovarian cancer has the highest mortality rate among all the reproductive cancers in women. Advanced stage diagnosis and chemo/radio-resistance is a major obstacle in treating advanced ovarian cancer. The most commonly employed chemotherapeutic drug for ovarian cancer treatment is cis-platin. As with most chemotherapeutic drugs, many patients eventually become resistant to cis-platin and therefore, diminishing its effect. The efficacy of current treatments may be improved by increasing the sensitivity of cancer cells to chemo/radiation therapies.

**Methods:**

The present study is focused on identifying the differential expression of regulatory microRNAs (miRNAs) between cis-platin sensitive (A2780), and cis-platin resistant (A2780/CP70) cell lines. Cell proliferation assays were conducted to test the sensitivity of the two cell lines to cis-platin. Differential expression patterns of miRNA between cis-platin sensitive and cis-platin resistant cell lines were analyzed using novel LNA technology.

**Results:**

Our results revealed changes in expression of 11 miRNAs out of 1,500 miRNAs analyzed. Out of the 11 miRNAs identified, 5 were up-regulated in the A2780/CP70 cell line and 6 were down regulated as compared to cis-platin sensitive A2780 cells. Our microRNA data was further validated by quantitative real-time PCR for these selected miRNAs. Ingenuity Pathway Analysis (IPA) and Kyoto Encyclopedia of Genes and Genomes (KEGG) analysis was performed for the selected miRNAs and their putative targets to identify the potential pathways and networks involved in cis-platin resistance.

**Conclusions:**

Our data clearly showed the differential expression of 11 miRNAs in cis-platin resistant cells, which could potentially target many important pathways including MAPK, TGF-β signaling, actin cytoskeleton, ubiquitin mediated proteasomal pathway, Wnt signaling, mTOR signaling, Notch signaling, apoptosis, and many other signaling pathways. Manipulation of one or more of these miRNAs could be an important approach for ovarian cancer chemotherapy.

## Background

Epithelial ovarian cancer (EOC) is the most common gynecologic malignancy and fifth most prevalent cancer in women worldwide [1]. According to cancer statistics, in the United States alone, 21,990 new cases of ovarian cancer will be diagnosed and approximately 15,460 of them will result in death in 2010 [2]. Despite advances in detection treatments, only 30% of patients with advanced stage ovarian cancer survive 5 years after initial diagnosis [3]. The high mortality rate is mainly attributable to late-stage diagnosis, lack of effective methods for the early diagnosis, and tumor resistance to chemotherapy. Genetic mutations have been studied which leads to chemotherapy resistance. Most notably, the BRCA1/2 mutations demonstrate a salient role in the pathogenesis of ovarian cancer resistance to chemotherapy [4]. More recently, epigenetic mechanisms like DNA methylation, histone modification, and recently microRNA regulation have been found to play an important role in the resistance of cancer cells to chemotherapeutic agents [5]. Interestingly, chemotherapy is the most viable and common treatment among the other treatments employed which include surgery and radiation therapy. Often treatments amalgamate multiple specialized chemotherapeutic drugs.

One such front-line chemotherapeutic drug for treating ovarian cancer is cis-platin. Cis-platin is an inorganic platinum-based compound formally named cis-diamminedichloroplatinum (II) (CDDP). Although, initially, this drug is successful in 80-90% of the patients, eventually cells become resistant [6,7]. Resistance to cis-platin occurs in nearly one third of all women during treatment and is prevalent in nearly all patients treated for a recurrent disease [8]. This leads to one question: what mechanisms cause cells to become resistant to cis-platin? Cis-platin reacts with DNA to induce distinctive biological changes that results in damaged DNA and starts the irrevocable apoptosis process [9]. When cis-platin penetrates cells its chloride channels are replaced by water molecules, forming aquated species that can react with intracellular macromolecules, creating cis-platin adducts. The presence of such adducts in DNA is thought to facilitate cell cycle arrest and apoptosis [10]. While several elements have been proposed as inducers for cis-platin resistance, the general consensus is cis-platin resistance results from multiple mechanisms, depending on the cell type [11]. Since 2006, much speculation has arisen on the correlation between miRNA, gene expression, and even carcinogenesis [12].

The role of microRNA (miRNA) in the molecular evolution of ovarian cancer has been of particular interest. miRNAs were formerly considered "junk" RNA. miRNAs are single stranded RNAs about 21-23 nucleotides long. Recent epigenetic studies support that these extremely short single-stranded RNAs have more impact than previously expected. Extensive research demonstrates that many genes are regulated by a single miRNA [13-15]. A possible link between miRNAs and cancer was first reported in chronic lymphocytic leukemia, where miR-15 and miR-16 were found to be down-regulated in a majority of the tumors [16]. Since then, as miRNAs have been associated with gene expression, investigators have begun conducting research on the relationship between miRNA and cancers [17-19]. The miRNA binds to semi-complimentary sites at the 3'-untranslated region of their targeted messenger RNA (mRNA), therefore suppressing the translation process [12]. This can result in one of two fates: mRNA degradation or translation truncation [20]. Therefore, miRNA can significantly affect gene expression. Because miRNAs are so critical in the post-transcriptional process, they could be used as potential therapeutic tools. Various investigations on specific miRNAs have exposed the functionality of select small RNAs [21-23]. The aim of this study is to determine any potential miRNA that could be linked to cis-platin resistance by identifying miRNA differences in cis-platin resistant and cis-platin sensitive cell lines.

## Methods

### Cell lines and cell culture

Human epithelial ovarian tumor cis-platin sensitive (A2780) cell line was obtained from Dr. Denise Connolly (Fox Chase Cancer Center, Philadelphia, PA). The cis-platin resistant (A2780/CP70) cell line was obtained from Dr. Christopher States (University of Louisville, Louisville, KY). A2780/CP70 cell line is derived from A2780 cell line and requires higher concentration of cis-platin to achieve cell death as compared to A2780 cells. Cell lines were cultured in RPMI 1640 supplemented with 10% fetal bovine serum and 1% antibiotics (Invitrogen, Carlsbad, CA) and maintained in a humidified atmosphere at 37°C and 5% CO_2_. The cell lines were sub-cultured on routinely basis every 3-4 days.

### Cell viability assays

A2780 and A2780/CP70 cell lines were cultured to test the responsiveness of each cell line to the cis-platin drug under our culture conditions. The cells were plated in 96 well plates (5,000 cells/well) as described previously [24]. After 24 h of plating, the cells medium was replaced with fresh medium containing 5% serum and six different concentrations of cis-platin (0, 2 μM, 20 μM, 40 μM, 100 μM and 200 μM). Cell viability assays were performed after 24 h, 48 h, and 72 h after treatment as described previously [24]. Briefly, medium in each well was replaced with fresh medium and MTT added in a ratio of 1:5 (Promega, Madison, WI). After two hours of incubation, absorbance was recorded using an ELISA plate reader at 492 nm.

### Extraction of miRNA

After 24 h of plating, cells were rinsed with PBS and total RNA from each sample was purified using miRNA Easy Mini Kit (QIAGEN, Valencia, CA). Total RNA was then quantited using NanoDrop.

### Integrity of miRNA

The quality of miRNA extracted was tested by using a Bioanalyzer (Agilent Technologies Preckel, Valer, Kratzmeier). The data retrieved from this analysis, projected the samples contained high levels of miRNA, which was applicable to our studies.

### Determination of specific miRNAs

miRNA analysis of three independent samples from each A2780 cell line and A2780/CP70 cell line respectively was performed in association with Exiqon Biotechnology Company (Copenhagen, Denmark). Analysis was performed using novel LNA technology. The miRNAs chips contained sequences from 1,500 known miRNAs. The hybridization, washing of non-specific RNAs, and comparative analysis of miRNAs was performed by Exiqon Biotechnology Company. The data was deposited to Array Express # E-MEXP-3141.

### Confirmation of miRNA expression

After evaluation of the Exiqon analysis, 11 miRNAs that were identified with different levels of expression between A2780 and A2780/CP70. Sequences of 5 miRNAs were commercially available; therefore, quantitative real-time PCR was performed on these 5 miRNAs which included miR-193b, miR-20b, miR625, let-7c, and miR-642. The miRNA kits for miR-193b, miR-20b, miR-625, let-7c, and miR-642 were purchased from Applied Biosystems (Foster City, CA) to quantitate their fold change in expression. For this purpose, total RNA was reverse transcribed using reverse transcription (RT/PCR) kits (Applied Biosystems) following the manufacturer's protocol. Briefly, miRNAs were reverse transcribed in a single reaction using 2 ml of each miRNA specific 5X RT primers. Resulting material was then used for independent qRT-PCR for each miRNA. Quantitative RT-PCR reactions were completed on a 7900 HT Sequence Detection System (Applied Biosystems). Samples were run in triplicate and the average values were used in subsequent analysis. Experiments were performed using at least 3 independent samples and data are displayed as mean ± SD.

### Statistical analysis

Data comparing differences in levels of expression of miRNAs between A2780 cis-platin sensitive and A2780/CP70 cis-platin resistant cell lines were analyzed using unpaired Student's t-test. Differences were considered significant when p < 0.05.

### Pathway analysis

The selected miRNAs were further analyzed to identify the networks and pathways targets. For this purpose, we used two independent software Ingenuity Pathway analysis (IPA) and Kyoto Encyclopedia of Genes and Genomes (KEGG). These pathways analysis software identified the putative targets for the input miRNA(s) and then developed the networks among the genes/targets.

## Results

### Cell viability assay

**Figure 1:**
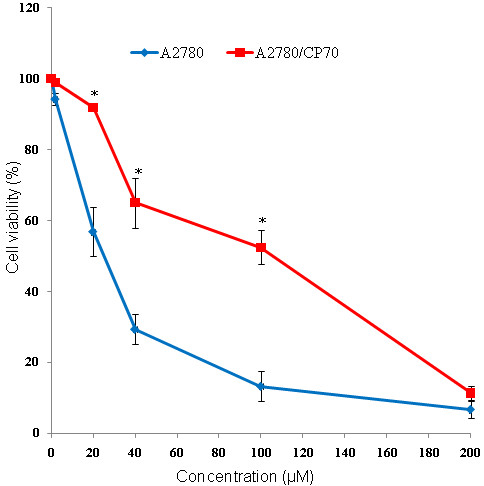
**Cell proliferation assay of A2780 and A2780/CP70 cells**. Viability of cells were assessed after 72 h in A2780 and A2780/CP70 cell lines post cis-platin treatment using MTT assay and showed that A2780/CP70 cells required 3 to 4-folds higher concentration of the cis-platin to achieve the same level of cell death compared to A2780 cells at 72 h. Error bars represent ± SEM (n = 3) of three independent experiments. * p-value ≤ 0.05.

To investigate the difference in the sensitivity of A2780 and A2780/CP70 cells for cis-platin, cell viability assays were performed. Our results showed that the A2780/CP70 cell line was significantly less sensitive to cis-platin compared to A2780 cell line (Figure 1). A2780/CP70 cells required 3 to 4-fold higher concentration of the cis-platin to achieve the same level of cell death compared to A2780 at 24 h, 48 h (data not shown), or 72 h of treatment (Figure 1), indicating reduced sensitivity of A2780/CP70 cells to cis-platin.

The quality of miRNA extracted was tested by using a Bioanalyzer. The double high peaks represent the successful extraction of RNA and integrity of RNA (results not shown). The major bands represent intensity of 28S and 18S ribosomal RNAs, two highly expressed control RNAs. The sharpness and peak reveal the quality of RNA. Based on these results, we concluded that a high quality of RNA was purified from each sample. High quality ribosomal RNAs lead to better quality of smaller size RNAs including miRNA. Further analysis showed that all samples had RNA integrity values of 8.9 or higher which are recommended for high quality array performance.

### miRNA comparison analysis

**Figure 2:**
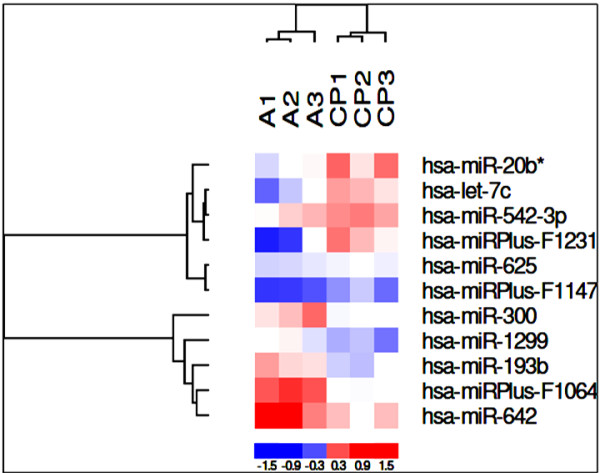
**Heat map diagram and hierarchical clustering of 11 miRNAs with different gene expression between A2780 and A2780/CP70 cell lines**.

miRNA analysis of the samples from A2780 and A2780/CP70 cell lines were screening for 1,500 miRNA sequences and a total of 11 miRNAs showed a difference in their expression levels between A2780 and A2780/CP70 cell lines. Figure 2 shows the result of the two-way hierarchical clustering of genes. Each row represents a miRNA, and each column represents a sample of either A2780 or A2780/CP70. The miRNA clustering tree is shown on the left. The clustering is performed on log2 (Hy3/Hy5) ratios which passed the filtering criteria on variation across the two sample groups with p-value < 0.05. The color scale shown at the bottom illustrates the relative expression level of a miRNA across all samples.

**Figure 3:**
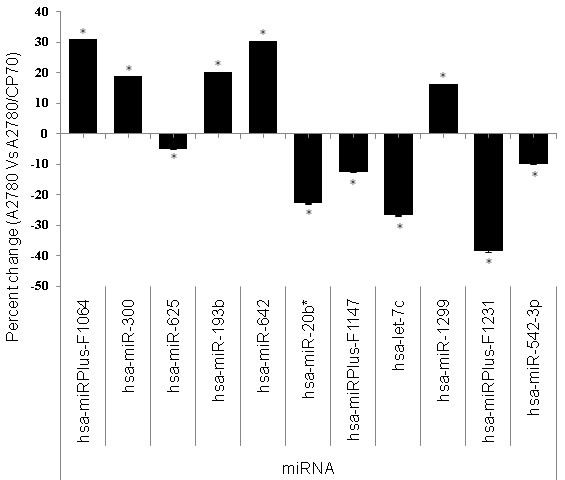
**Microarray analysis of differentially expressed miRNAs between A2780 and A2780/CP70 ovarian cancer cell lines**. The bars represents normalized % change values with mean ± SD (n = 3) between A2780 and A2780/CP70. * represents significant at p-value (≤ 0.05). The data presented show that miRNAs hsa-miRPlus-F1064, hsa-miR-300, hsa-miR-193b, hsa-miR-642 and hsa-miR-1299 were upregulated 32%, 18%, 19%, 29% and 16% respectively, whereas hsa-miR-625, hsa-miR-20b, hsa-miRPlus-F1147, hsa-let-7c, hsa-miRPlus-F1231, and hsa-miR-542-3p were down regulated 4%, 23%, 12%, 28%, 38% and 10% respectively in A2780/CP70 cells as compared to A2780 cells.

**Figure 4:**
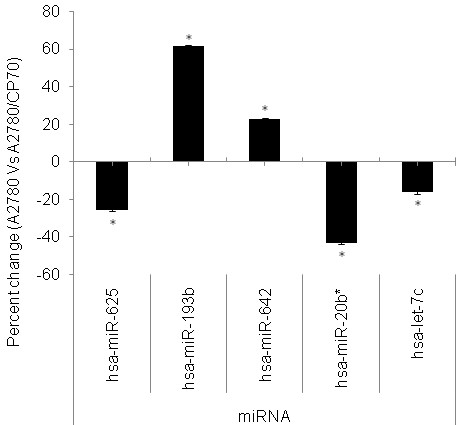
**Quantitative real-time PCR (qRT-PCR) analysis of differentially expressed miRNAs in A2780 vs. A2780/CP70 ovarian cancer cell lines**. Cells were harvested for total RNA and subjected to cDNA synthesis. Expression levels of 5 miRNAs were analyzed by qRT-PCR. The bars represents normalized % change values with mean ± SD (n = 3) between A2780 and A2780/CP70. *represents significant at p-value (≤ 0.05). The data presented showed that miRNAs hsa-miR-193b and hsa-miR-642 were upregulated 64% and 22% respectively, whereas hsa-miR-625, hsa-miR-20b, and hsa-let-7c were down regulated 22%, 44% and 18% respectively in A2780/CP70 cells as compared to A2780 cells.

Figure 3 is a graphical representation of the up-regulation and down-regulation of miRNAs demonstrated in Figure 2 and corresponds to the percent change in expression of miRNAs in A2780 and A2780/CP70 cell lines. Out of 11 miRNAs that showed differential expression, 5 were up-regulated and 6 were down regulated in A2780/CP70 cell line compared to A2780 cell line. Up-regulated miRNAs include hsa-miRplus-F1064, hsa-miR-300, hsa-miR-193b, hsa-miR-642, and hsa-miR-1299. Out of 11 miRNA, 6 were down-regulated: hsa-miR-625, hsa-miR-20b, hsa-miRPlus-F1147, hsa-let-7c, hsa-miRPlus-F1231, and hsa-miR-542-3p. Hsa-miRPlus-F1064 was the highest up-regulated miRNA (30%), while hsa-miRPlus-F1231 was significantly down regulated (38%). Out of the 11 miRNAs, 5 were tested using qRT-PCR. The results revealed similar patterns of differential expression as analyzed by miRNA array (Figure 4).

The IPA and KEGG pathway analysis software revealed that out of 7 miRNAs selected for analysis, most of them including miR-20b (32 genes), miR-300 (24 genes), let-7c (22 genes), miR-193b (8 genes), miR-542-3p (7 genes) and miR-642 (4 genes) target MAPK signaling pathway (Additional file 1). MAPK signaling pathway was the most affected pathway by these miRNAs with total of 73 genes affected by 7 selected miRNAs, with the greatest affect by miR-20b and let-7c (Additional file 1).

TGF-β signaling pathway, actin cytoskeleton, ubiquitin mediated proteolysis, Wnt signaling, mTOR signaling, Notch signaling, and apoptosis are few other important pathways affected by these miRNAs (Additional file 1). Among them TGF-β signaling, Wnt signaling, ubiquitin mediated proteolysis, and Notch signaling are top most signaling pathways affected by miR-300 (Additional file 2), whereas ubiquitin proteolysis, p53 signaling, and mTOR signaling are a few of the important signaling pathways affected by miR-625 (Table 1).

**Table 1:** Pathways affected by the putative targets of miR-625

KEGG Pathway	Gene Name	Found Genes	-ln(p-value)	KEGG Pathway ID
Ubiquitin mediated proteolysis	UBE2NL, UBE2N, FBXW7, SIAH1	4	12.75	hsa04120

p53 signaling pathway	SIAH1, IGF1	2	4.77	hsa04115

Regulation of autophagy	ATG5	1	1.62	hsa04140

Focal adhesion	COL1A1, IGF1	2	1.27	hsa04510

Neurodegenerative Diseases	FBXW7	1	1.16	hsa01510

Lysine degradation	SETD1A	1	1.16	hsa00310

mTOR signaling pathway	IGF1	1	0.9	hsa04150

Glioma	IGF1	1	0.59	hsa05214

Glycerophospholipid metabolism	ACHE	1	0.55	hsa00564

Renal cell carcinoma	ARNT	1	0.52	hsa05211

Melanoma	IGF1	1	0.49	hsa05218

Long-term depression	IGF1	1	0.46	hsa04730

ECM-receptor interaction	COL1A1	1	0.4	hsa04512

Hematopoietic cell lineage	CSF3R	1	0.39	hsa04640

Neuroactive ligand-receptor interaction	GHRHR	1	0.37	hsa04080

Cytokine-cytokine receptor interaction	CSF3R	1	0.37	hsa04060

Prostate cancer	IGF1	1	0.32	hsa05215

TGF-beta signaling pathway	FST	1	0.31	hsa04350

Axon guidance	SEMA6C	1	0.1	hsa04360

Cell Communication	COL1A1	1	0.04	hsa01430

Jak-STAT signaling pathway	CSF3R	1	0.01	hsa04630

Wnt signaling pathway	SIAH1	1	0	hsa04310

**Figure 5:**
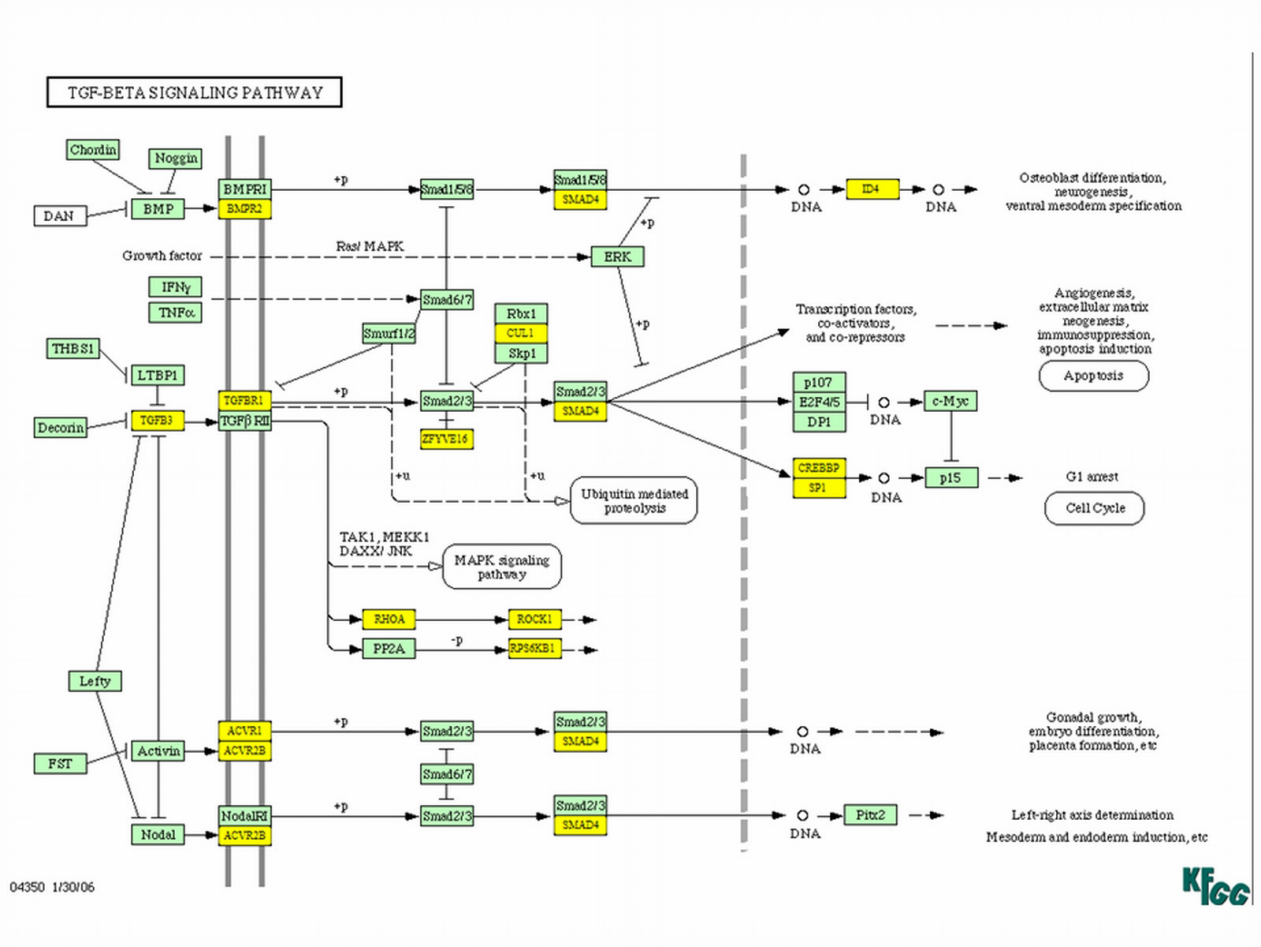
**Effect of miR-300 on TGF-β signaling pathway**. Kyoto Encyclopedia of Genes and Genomes (KEGG) analysis was used to identify the putative targets (yellow) for miR-300.

**Figure 6:**
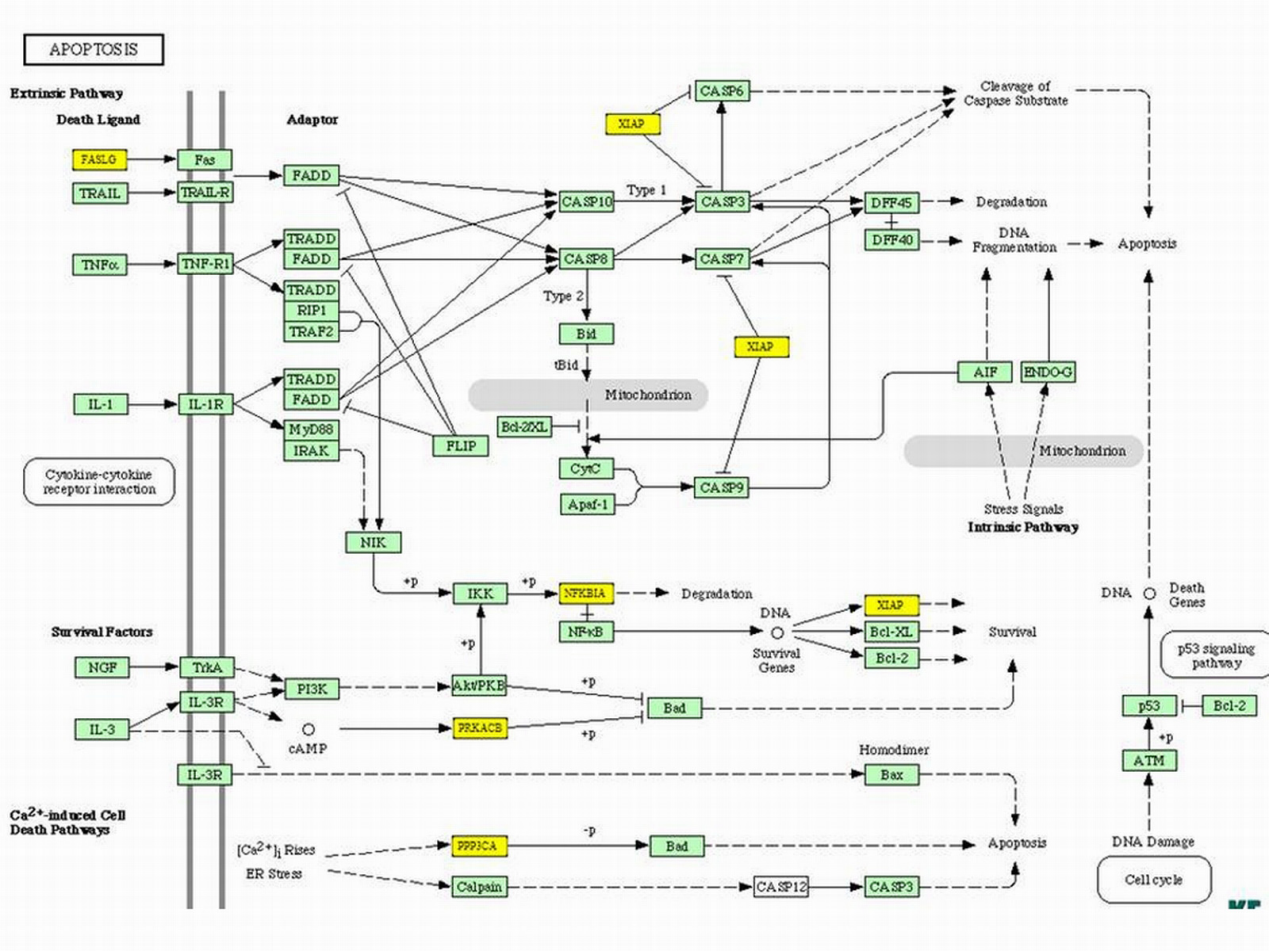
**Influence of miR-300 on apoptosis pathway**. Kyoto Encyclopedia of Genes and Genomes (KEGG) analysis was used to identify the putative targets (yellow) for miR-300.

**Figure 7:**
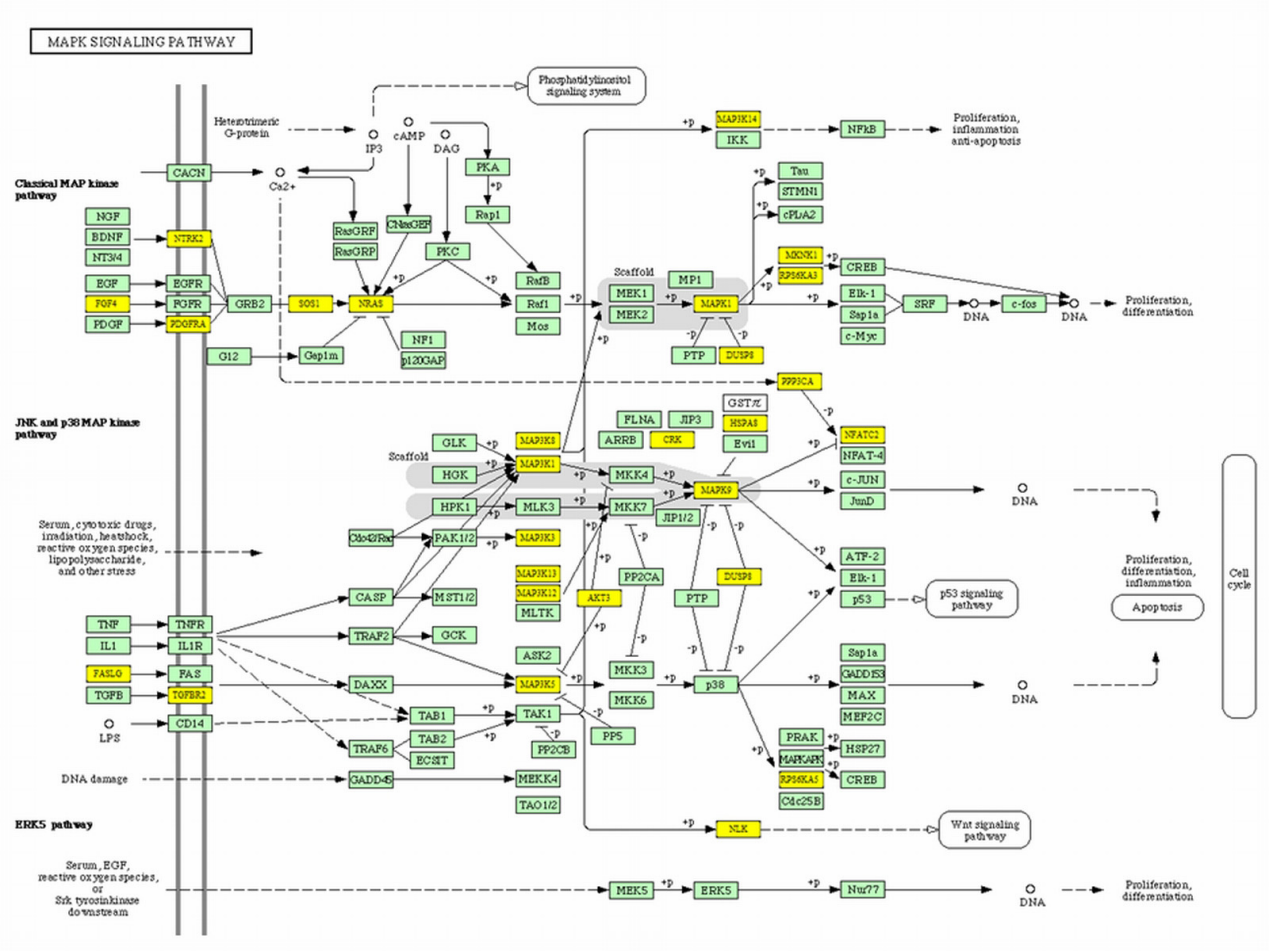
**MAPK signaling and miR-20b**. Kyoto Encyclopedia of Genes and Genomes (KEGG) analysis was used to identify the putative targets (yellow) for miR-20b.

**Figure 8:**
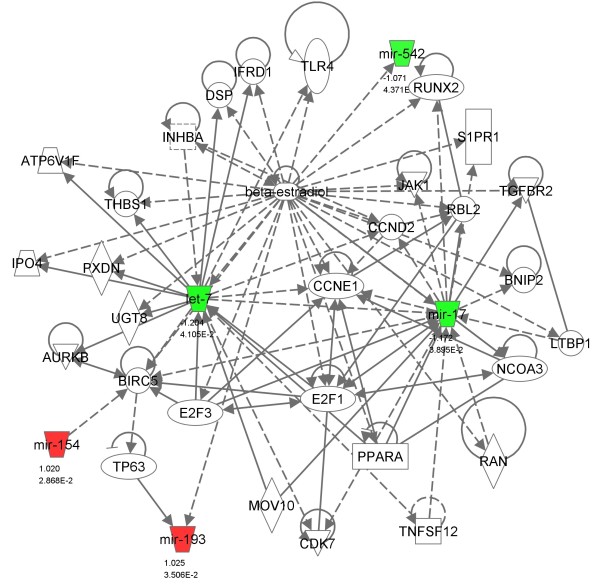
**Effect of miRNAs on cell cycle, proliferation, and differentiation**. Selected miRNAs that are differentially expressed in cis-platin resistant cells were used to generate networks using Ingenuity Pathway Analysis (IPA) software. miRNAs shown in green are down-regulated and those in red were up-regulated. The genes connected with dotted lines were those affected indirectly, and the ones connected with solid lines are the ones affected directly.

When we analyzed the genes affected by miR-300 in TGF-β signaling, TGF-β itself along with its receptor TGFβR1 and other downstream molecules such as SMAD4, CREBP, and SP1 were targeted by miR-300 (Figure 5). KEGG analysis also revealed that miR-300 affects apoptosis by targeting FAS ligand, NF-κB, and other proteins (Figure 6). Similarly, insulin like growth factor-1 (IGF-1) and seven in absentia homolog 1 (SIAH1) are the genes targeted by miR-625 in p53 signaling pathway (Table 1). Among the miRNAs analyzed, miR-20b targets highest number of genes in MAPK signaling pathway (32 genes) which includes FAS ligand, FGF4, TGF-β receptor 2 (TGFβR2), and various MAP kinases (Figure 7). Whereas the IPA analysis showed that let-7c targets many genes directly (solid lines) or indirectly (dotted lines) including transcriptional factor E2F3, cyclin-dependent kinase-7, PPAR-α, TWEAK (Tnfsf12), cyclin D2, cyclin E1, β-estradiol pathway as well as many other genes (Figure 8).

## Discussion

Epithelial ovarian cancer (EOC) is the most fatal gynecologic malignancy [25]. The high mortality rate is due to late diagnosis, as epithelial ovarian tumors commonly lack early symptoms, as well as development of chemo-resistance during treatment. So far, many attempts have been made to predict the biology of ovarian tumors to determine the prognosis and to develop new therapeutic strategies. With the advent of miRNA technology in recent years, it is now possible to expand our knowledge to better understand ovarian cancer by analyzing miRNA mediated pathways. Several recent studies indicate that miRNA have altered expression pattern in ovarian cancer [18,26-28].

Chemotherapy is the preferred treatment for malignancies. However, a successful long-term use of chemotherapy is often prevented by the development of drug resistance. Drug resistance was first documented experimentally in mouse leukemic cells that acquired resistance to methotrexate in a laboratory model in 1950, indicating that drug resistance is the main cause of treatment failure [29]. So far studies have indicated that there are significant differences in miRNA expression pattern between chemotherapeutic sensitive and resistant ovarian cancer cell lines and tissues. Boren et al. [30] reported 27 miRNAs that were related to ovarian cancer cell line sensitivity to platinum-based chemotherapeutic agents. Similarly, Eitan et al. [31] reported several miRNAs that were differentially expressed in stage 3 ovarian tumors. The difference in miRNA expression pattern between chemotherapy sensitive and resistant cells will prove to be clinically significant.

The main purpose of our study was to determine the miRNA differences between cis-platin sensitive A2780 and resistant A2780/CP70 cell lines. It was hypothesized that the two cell lines would exhibit differences in miRNA expression pattern. Our results demonstrated that 11 miRNAs are differentially expressed in A2780/CP70 cell line compared to A2780 cell line. Recently, White et al. [32] compiled data from eight published studies and reported several dysregulated miRNAs in ovarian cancer. Yang et al. [33] reported that let-7i expression was significantly reduced in chemotherapy-resistant ovarian cancer patients and lower level of expression of let-7i is strongly associated with shorter progression-free survival. Sorrentino et al. [34] analyzed the miRNA profile in a panel of paclitaxel resistant (A2780TAX, A2780TC1 and A2780TC3) and cis-platin resistant (A2780CIS) cell lines and reported down regulation of miRNA-30c, miRNA-130a, and miRNA-335 in all the resistant cell lines, suggesting a direct involvement of these miRNAs in the development of chemoresistance. Our data suggests that the 5 up-regulated miRNAs and the 6 down-regulated miRNAs found in the A2780/CP70 ovarian cancer cell lines could contribute to the sensitivity of ovarian cancer cells. Out of these 11 differentially expressed miRNAs 5 were validated by qRT-PCR which showed directional correspondence with our microRNA data.

KEGG analysis of selected miRNAs which showed differential expression in cis-platin resistant cells and further validated in qRT-PCR revealed that these miRNAs have putative targets involved in many important pathways including TGF-β, apoptosis, p53, MAPK, IGF, and other signaling pathways. MAPK signaling is the most affected pathway by these 5 miRNAs, out of which, miR-20b has the highest target score and number for its potential putative targets (Figure 7). Exact mechanism(s) by which cis-platin attains its anticancer function are unknown, however, activation of apoptotic pathway via MAPK signaling is one of its major mechanisms of action [35]. Activation of MAPK via phosphorylation can lead to either cell proliferation or apoptosis. The KEGG analysis of miR-20b showed that there are many putative targets for miR-20b involved in MAPK signaling (Figure 7). Genes including FAS ligand G (FASLG), FGF4, DUSP8, MAPK1, TGFβR2) and various MAP3Ks are found to be putative targets for miR-20b. Our miRNA analysis showed that miR-20b was down-regulated, which is further validated by qRT-PCR. Therefore, in conjunctions with our data and other published reports, it may be possible that the cis-platin resistance in the cells can be due to the down-regulation of miR-20b, which could potentially target genes like DUSP8 and thereby inhibit p38 and MAPK9 axis for apoptosis (Figure 8). These findings are further supported by recent studies by Wang et al. [36], who showed that MAPK signaling is important for cis-platin induced cell death. In addition to FAS ligand G (FASLG), miR-300 can also target NF-κB, PRKACB and other proteins involved in apoptosis pathway (Figure 6). This information further support the notion that up-regulation of miR-300 promoting cis-platin resistance in the cells by targeting many genes involved in apoptosis and cell cycle.

TGF-β signaling is the second most affected pathway by these miRNAs (Additional file 1). We also observed that miR-300 has the highest number of putative targets involved in this pathway. TGF-β is involved in cell proliferation, cell adhesion, cell migration, and cell differentiation [37] and is up-regulated in many tumors [38]. Although not much is known about its role in cis-platin induced cell death, but recent evidences suggest that decreased expression of TGFβR1 is observed in cis-platin and TGF-β resistant L1210 cells [39]. In addition down-regulation of Smad proteins could induce cis-platin resistance [40]. Our miRNA array showed the up-regulation of miR-300, which can potentially target genes including TGFβR1 and many Smad proteins (Figure 5). From these observations, the cis-platin resistance in these cells may be mediated through induction of miR-300 which may regulate TGF-β induced apoptosis and cell cycle.

Ingenuity Pathway Analysis (IPA) of selected miRNAs showed that let-7 is involved in regulation of cell cycle, growth, proliferation and differentiation (Figure 8). Genes affected by let-7 are indirectly connected with dotted lines, whereas the genes connected with solid lines are affected directly. According to IPA, let-7 decreases the expression of cyclin-dependent kinase 7 (Cdk7) [41]. Cell cycle-dependent kinases are important for cell division, and inhibitors of cdk are found to be involved in improving sensitivity to cis-platin [42]. IPA also showed that let-7 decreases the expression of cyclin D and E [41]. Our miRNA array showed the down-regulation of let-7, which is further validated by qRT-PCR. From these observations, one of the potential mechanisms of cis-platin resistance to these cells may be result of down-regulation of let-7, which could be an effective inhibitor of Cdk7.

Thus, in theory, if the expression of these miRNAs is reversed in A2780/CP70; these cells should become vulnerable to cis-platin. The cell viability test supported that the A2780 cell line is more susceptible to cis-platin. Consistent with our findings, Parker et al. [43], using A2780 and A2780/CP70 cell lines studied their respective characteristics of drug accumulation and efflux, cytosolic inactivation of drug, and DNA repair, showed that the A2780/CP70 cell line was 13-fold more resistant to cis-platin than A2780 cells.

The A2780/CP70 cell line demonstrated being more resistant to cis-platin and revealed differential expression of 11 miRNAs. Even though difference in the levels of these 11 miRNAs between two cell lines is moderate but could be highly significant to change the sensitivity of ovarian cancer to cis-platin. Therefore, defining the function of miRNAs that are differentially expressed in A2780 and A2780/CP70 cell lines identified in our studies could be highly significant in relation to change in sensitivity of A2780 cell line to cis-platin, which could lead to better management of cis-platin resistance ovarian cancer.

## Conclusions

Identification of the differential miRNA expression pattern in human EOCs towards the resistance to cis-platin, as well as their targets in case of ovarian cancer, provides new opportunities for therapeutic strategies. miRNA-based gene therapy targeting deregulated miRNAs will be a future tool for cancer diagnosis and treatment. Cis-platin resistance can significantly impede a patient's survival and recovery chances. Our study has taken a step to identify the differential miRNA expression in two cell lines to potentially re-sensitize cis-platin resistant cells. The KEGG and IPA analysis of the selected miRNAs clearly showed that the differentially expressed miRNAs affected many important pathways including TGF-β, apoptosis, MAPK, p53 and many other signaling pathways, which have direct or indirect role in cis-platin mediated cell death. Detailed understanding of the characteristic miRNA abnormalities could contribute to novel approaches in early diagnosis and better management of ovarian cancer.

## Conflict of interests statement

The authors declare that they have no competing interests.

## Authors' contributions

SK, AK, and PS performed experiments and were responsible for data collection, analysis, and interpretation of the results. SKP performed pathway analysis, target search, and network development. SK, AK, PS drafted the manuscript, and SKP has provided important input in writing the manuscript. SNR was involved in statistical analysis of the data. SSK was responsible for experimental design, providing the proper directions to the study, and critically revising the manuscript. All authors have read and approved the final manuscript.

## Supplementary Material

Additional file 1 **List of pathways affected by the targets of selected miRNAs**. We used KEGG pathway analysis to identify the targets for the selected miRNAs from our analysis and the pathways affected by these targets. The p-values given for each miRNA corresponds to the number of targets involved in that particular pathway against total number of molecules or genes present in each pathway. Click here for file

Additional file 2 **List of pathways affected by the targets of miR-300**. We used KEGG pathway analysis to identify the targets for the miR-300 and the pathways affected. Found genes column represents the number of miR-300 targets present in a particular pathway. The p-values given correspond to the number of targets involved in that particular pathway against total number of molecules or genes present in each pathway. Click here for file

## References

[B1] Aletti GD Gallenberg MM Cliby WA Jatoi A Hartmann LC Current management strategies for ovarian cancer Mayo Clin Proc 2007 82 751 770 10.4065/82.6.751 17550756

[B2] Siegel R Ward E Brawley O Jemal A Cancer Statistics CA Cancer J Clin 2011 61 212 236 10.3322/caac.20121 21685461

[B3] Cannistra SA Cancer of the ovary N Engl J Med 2004 351 24 2519 2529 10.1056/NEJMra041842 15590954

[B4] Swisher EM Sakai W Karlan BY Wurz K Urban N Taniguchi T Secondary BRCA1 mutations in BRCA1-mutated ovarian carcinomas with platinum resistance Cancer Res 2008 68 2581 2586 10.1158/0008-5472.CAN-08-0088 18413725 PMC2674369

[B5] Balch C Fang F Matei DE Huang TH Nephew KP Minireview: epigenetic changes in ovarian cancer Endocrinology 2009 150 4003 4011 10.1210/en.2009-0404 19574400 PMC2736079

[B6] Borst P Rottenberg S Jonkers J How do real tumors become resistant to cisplatin? Cell Cycle 2008 7 1353 1359 10.4161/cc.7.10.5930 18418074

[B7] Van Jaarsveld MT Helleman J Berns EM Wiemer EA MicroRNAs in ovarian cancer biology and therapy resistance Int J Biochem Cell Biol 42 1282 1290 10.1016/j.biocel.2010.01.014 20083225

[B8] Zamble DB Lippard SJ Cisplatin and DNA repair in cancer chemotherapy Trends Biochem Sci 1995 20 435 439 10.1016/S0968-0004(00)89095-7 8533159

[B9] Siddik ZH Cisplatin: mode of cytotoxic action and molecular basis of resistance Oncogene 2003 22 47 7265 7279 10.1038/sj.onc.1206933 14576837

[B10] Kartalou M Essigmann JM Mechanisms of resistance to cisplatin Mutat Res 2001 478 23 43 10.1016/S0027-5107(01)00141-5 11406167

[B11] Wu C Wangpaichitr M Feun L Kuo MT Robles C Lampidis T Savaraj N Overcoming cisplatin resistance by mTOR inhibitor in lung cancer Mol Cancer 2005 4 25 10.1186/1476-4598-4-25 16033649 PMC1181826

[B12] Mezzanzanica D Bagnoli M De Cecco L Valeri B Canevari S Role of microRNAs in ovarian cancer pathogenesis and potential clinical implications Int J Biochem Cell Biol 42 1262 1272 10.1016/j.biocel.2009.12.017 20035894

[B13] Bartel DP MicroRNAs: genomics, biogenesis, mechanism, and function Cell 2004 116 2 281 297 10.1016/S0092-8674(04)00045-5 14744438

[B14] Ambros V The functions of animal microRNAs Nature 2004 431 7006 350 355 10.1038/nature02871 15372042

[B15] Cannell IG Kong YW Bushell M How do microRNAs regulate gene expression? Biochem Soc Trans 2008 36 1224 1231 10.1042/BST0361224 19021530

[B16] Calin G Frequent deletions and down-regulation of micro-RNA genes miR15 and miR16 at 13q14 in chronic lymphocytic leukemia Proc Natl Acad Sci USA 2002 99 15524 15529 10.1073/pnas.242606799 12434020 PMC137750

[B17] Lu J Getz G Miska EA Alvarez-Saavedra E Lamb J Peck D Sweet-Cordero A Ebert BL Mak RH Ferrando AA Downing JR Jacks T Horvitz HR Golub TR MicroRNA expression profiles classify human cancers Nature 2005 435 834 838 10.1038/nature03702 15944708

[B18] Iorio M Visone R Di Leva G Donati V Petrocca F Casalini P Taccioli C Volinia S Liu C Alder H Calin GA Menard S Croce CM MicroRNA signatures in human ovarian cancer Cancer Res 2007 67 8699 8707 10.1158/0008-5472.CAN-07-1936 17875710

[B19] Taylor DD Gercel-Taylor C MicroRNA signatures of tumor-derived exosomes as diagnostic biomarkers of ovarian cancer Gynecol Oncol 2008 110 13 21 10.1016/j.ygyno.2008.04.033 18589210

[B20] Yang H Kong W He L Zhao JJ O'Donnell JD Wang J Wenham RM Coppola D Kruk PA Nicosia SV Cheng JQ MicroRNA expression profiling in human ovarian cancer: miR-214 induces cell survival and cisplatin resistance by targeting PTEN Cancer Res 2008 68 425 433 10.1158/0008-5472.CAN-07-2488 18199536

[B21] Johnson SM Grosshans H Shingara J Byrom M Jarvis R Cheng A Labourier E Reinert KL Brown D Slack FJ RAS is regulated by the let-7 microRNA family Cell 2005 120 5 635 647 10.1016/j.cell.2005.01.014 15766527

[B22] Metzler M Wilda M Busch K Viehmann S Borkhardt A High expression of precursor microRNA-155/BIC RNA in children with Burkitt lymphoma Genes Chromosomes Cancer 2004 39 167 169 10.1002/gcc.10316 14695998

[B23] Zhu H Wu H Liu X Evans BR Medina DJ Liu CG Yang JM Role of MicroRNA miR-27a and miR-451 in the regulation of MDR1/P-glycoprotein expression in human cancer cells Biochem Pharmacol 2008 76 582 588 10.1016/j.bcp.2008.06.007 18619946 PMC2628586

[B24] Hamid T Malik MT Kakar SS Ectopic expression of PTTG1/securin promotes tumorigenesis in human embryonic kidney cells Mol Cancer 2005 4 3 10.1186/1476-4598-4-3 15649325 PMC546418

[B25] Sankaranarayanan R Ferlay J Worldwide burden of gynaecological cancer: the size of the problem Best Pract Res Clin Obstet Gynaecol 2006 20 207 225 10.1016/j.bpobgyn.2005.10.007 16359925

[B26] Corney DC Nikitin AY MicroRNA and ovarian cancer Histol Histopathol 2008 23 9 1161 1169 18581287 10.14670/hh-23.1161 PMC2691660

[B27] Shah PP Hutchinson LE Kakar SS Emerging role of microRNAs in diagnosis and treatment of various diseases including ovarian cancer J Ovarian Res 2009 2 11 10.1186/1757-2215-2-11 19712461 PMC2744658

[B28] Dahiya N Morin PJ MicroRNAs in ovarian carcinomas Endocr Relat Cancer 17 F77 89 10.1677/ERC-09-0203 PMC2856347 19903743

[B29] Burchenal JH Robinson E Johnston SF Kushida MN The induction of resistance to 4-amino-N10-methylpteroylglutamic acid in a strain of transmitted mouse leukemia Science 1950 111 116 10.1126/science.111.2875.116 15400457

[B30] Boren T Xiong Y Hakam A Wenham R Apte S Chan G Kamath SG Chen DT Dressman H Lancaster JM MicroRNAs and their target messenger RNAs associated with ovarian cancer response to chemotherapy Gynecol Oncol 2009 113 249 255 10.1016/j.ygyno.2009.01.014 19237188

[B31] Eitan R Kushnir M Lithwick-Yanai G David MB Hoshen M Glezerman M Hod M Sabah G Rosenwald S Levavi H Tumor microRNA expression patterns associated with resistance to platinum based chemotherapy and survival in ovarian cancer patients Gynecol Oncol 2009 114 253 259 10.1016/j.ygyno.2009.04.024 19446316

[B32] White NM Chow TF Mejia-Guerrero S Diamandis M Rofael Y Faragalla H Mankaruous M Gabril M Girgis A Yousef GM Three dysregulated miRNAs control kallikrein 10 expression and cell proliferation in ovarian cancer Br J Cancer 102 1244 1253 10.1038/sj.bjc.6605634 PMC2856011 20354523

[B33] Yang N Kaur S Volinia S Greshock J Lassus H Hasegawa K Liang S Leminen A Deng S Smith L Johnstone CN Chen XM Liu CG Huang Q Katsaros D Calin GA Weber BL Butzow R Croce CM Coukos G Zhang L MicroRNA microarray identifies Let-7i as a novel biomarker and therapeutic target in human epithelial ovarian cancer Cancer Res 2008 68 10307 10314 10.1158/0008-5472.CAN-08-1954 19074899 PMC2762326

[B34] Sorrentino A Liu CG Addario A Peschle C Scambia G Ferlini C Role of microRNAs in drug resistant ovarian cancer cells Gynecol Oncol 2008 111 478 486 10.1016/j.ygyno.2008.08.017 18823650

[B35] Wang D Lippard SJ Cellular processing of platinum anticancer drugs Nat Rev Drug Discov 2005 4 307 320 10.1038/nrd1691 15789122

[B36] Wang Z Xu J Zhou JY Liu Y Wu GS Mitogen-Activated Protein Kinase Phosphatase-1 Is Required for Cisplatin Resistance Cancer Res 2006 66 8870 8877 10.1158/0008-5472.CAN-06-1280 16951204

[B37] Kim JE Kim SJ Lee BH Park RW Kim KS Kim IS Identification of motifs for cell adhesion within the repeated domains of transforming growth factor-beta-induced gene, betaig-h3 J Biol Chem 2000 275 30907 15 10906123 10.1074/jbc.M002752200

[B38] Irigoyen M Pajares MJ Agorreta J Ponz-Sarvisé M Salvo E Lozano MD Pío R Gil-Bazo I Rouzaut A TGFBI expression is associated with a better response to chemotherapy in NSCLC Mol Cancer 2010 9 130 10.1186/1476-4598-9-130 20509890 PMC2900244

[B39] Stoika R Yakymovych M Souchelnytskyi S Yakymovych I Potential role of transforming growth factor beta1 in drug resistance of tumor cells Acta Bioch Polonica 2003 50 2 497 508 12833174

[B40] Xu S Xue C Li J Bi Y Cao Y Mare's disease virus type 1 microRNA miR-M3 suppresses cisplatin-induced apoptosis by targeting Smad2 of the transforming growth factor beta signal pathway J Viral 2011 85 276 85 10.1128/JVI.01392-10 PMC3014179 20962090

[B41] Buenos MJ Gomez de Cordon M Laresgoiti U Fernández-Piqueras J Zubiaga AM Malumbres Multiple E2F-Induced MicroRNAs Prevent Replicative Stress in Response to Mitogenic Signaling Mol Cell Biol 2010 30 2983 2995 10.1128/MCB.01372-09 20404092 PMC2876680

[B42] Wei J Zhao J Long M Han Y Wang X Lin F Ren J He T Zhang H p21WAF1/CIP1 gene transcriptional activation exerts cell growth inhibition and enhances chemosensitivity to cisplatin in lung carcinoma cell BMC Cancer 2010 10 632 10.1186/1471-2407-10-632 21087528 PMC2995802

[B43] Parker RJ Eastman A Bostick-Bruton F Reed E Acquired cisplatin resistance in human ovarian cancer cells is associated with enhanced repair of cisplatin-DNA lesions and reduced drug accumulation J Clin Invest 1991 87 772 777 10.1172/JCI115080 1999494 PMC329864

